# eIF4GI Facilitates the MicroRNA-Mediated Gene Silencing

**DOI:** 10.1371/journal.pone.0055725

**Published:** 2013-02-07

**Authors:** Incheol Ryu, Ji Hoon Park, Sihyeon An, Oh Sung Kwon, Sung Key Jang

**Affiliations:** 1 Molecular Virology Laboratory, POSTECH Biotech Center, Department of Life Science, Pohang University of Science and Technology, Pohang, Korea; 2 Division of Integrative Biosciences and Biotechnology, Pohang University of Science and Technology, Pohang, Korea; 3 Biotechnology Research Center, Pohang University of Science and Technology, Pohang, Korea; National Institute of Health, United States of America

## Abstract

MicroRNAs (miRNAs) are small noncoding RNAs that mediate post-transcriptional gene silencing by binding to complementary target mRNAs and recruiting the miRNA-containing ribonucleoprotein complexes to the mRNAs. However, the molecular basis of this silencing is unclear. Here, we show that human Ago2 associates with the cap-binding protein complex and this association is mediated by human eIF4GI, a scaffold protein required for the translation initiation. Using a cap photo-crosslinking method, we show that Ago2 closely associates with the cap structure. Taken together, these data suggest that eIF4GI participates in the miRNA-mediated post-transcriptional gene silencing by promoting the association of Ago2 with the cap-binding complex.

## Introduction

MicroRNAs (miRNAs), which play pivotal roles in numerous biological processes such as development, differentiation, proliferation, apoptosis, metabolic control, etc., are known to mediate the post-transcriptional gene silencing in various ways [1]. Many miRNAs degrade the targeted mRNAs by promoting their deadenylation and/or decapping, resulting in the repression of gene expression [2]. Also, many reports have indicated that miRNAs participate in the gene silencing by decreasing the translation of mRNAs. Several studies have suggested that miRNAs can reduce translation of their target mRNAs at the post-initiation stage (i.e., the elongation step), based on observations that miRNAs co-migrate with polyribosomes and their polysomal distributions are not altered during the gene repression [3]–[5]. However, recent studies have suggested that the translational repression by miRNAs occurs at the initiation step of translation, as indicated by the finding that mRNAs containing the 7-methyl guanosine cap structure at their 5′ ends (5′ cap structure), but not uncapped or internal ribosome entry site (IRES)-containing mRNAs, respond to the miRNA-mediated translational repression [6]–[10]. Some reports have suggested that the poly(A) tail at the 3′ end of mRNA is also involved in the translational repression. However, it still remains obscure whether the poly(A) tail is essential for the translational repression since the mRNA containing the 3′ histone step-loop instead of the 3′ poly(A) tail undergoes the translational repression by miRNAs [11], [12].

The 5′ cap structure of a cellular mRNA plays a critical role in cap-dependent translation, which is directed by the eIF4F complex; this complex is composed of eIF4E, which recognizes the cap structure, eIF4A, which is an RNA helicase, and eIF4G, which is a scaffold protein that interacts with many initiation factors (e.g., eIF4E, eIF4A, PABP and eIF3) and then with the 40S ribosome [13]. Several reports found that Argonaute (Ago) protein families such as MILI, PIWI, human and *Drosophila* Ago proteins, etc. associate with the cap-binding complexes [11], [14]–[18], suggesting that the miRNA-containing silencing complex could communicate with the cap-binding complex to induce the post-transcriptional gene silencing. To explain the necessity of the cap structure for the translational repression, the idea of ‘cap-competition’ by the Ago proteins has been proposed [19]. It suggests that human Argonaute 2 (Ago2) induces the post-transcriptional gene silencing by competing with eIF4E for an interaction with the 5′ cap structure of the target mRNA through its putative cap binding-like motif called the MC domain. Additionally, it was reported that *Drosophila* Argonaute 1 (dAgo1) directly binds the cap structure through its MID domain, which was allosterically regulated by miRNAs [17]. However, other reports have provided controversial evidences against the hypothesis. For instance, mutation of the phenylalanines in dAgo1 equivalent to those proposed to be required for cap-binding by human Ago2 does not impair its cap-binding ability, but rather abrogates its interactions with both miRNAs and GW182 [11]. Moreover, computational studies on the MC region of human Ago2 have indicated that it does not contain an eIF4E-like cap-binding motif and, furthermore, that the key aromatic residues (F470 and F505) are buried in the hydrophobic parts of the protein rather than exposed on the surface, creating an unfavorable configuration for their interactions with the cap structure [20]. Finally, a study from the soluble structure of the MID domain of human Ago2 showed that it binds to cap analogs nonspecifically [21]. The analysis of the crystal structure of human full-length Ago2 also confirms that the F470 and F505 contribute to the hydrophobic core of the MID domain [22].

Here, we investigated the putative association between Ago and the cap-binding complex and identified a translation factor that facilitates this association. We observed that Ago2 associates with the cap-binding protein complex in human cells and that ectopic expression of eIF4GI in cells stimulates the Ago2-cap association. Finally, we showed that Ago2 either interacts directly with or resides very close to the 5′ cap structure by using UV crosslinking experiments with RNAs carrying the [α^32^P]-labeled 5′-cap moiety. The data suggest that a translation initiation factor eIF4GI mediates a functional communication between the 5′ cap structure-associated eIF4F complex and the miRNA-containing silencing complex.

## Materials and Methods

### Construction of Plasmids

To generate pcDNA3.1-Flag or pcDNA3.1-myc, oligonucleotides encoding the Flag tag (Flag-sense and Flag-antisense) or the myc tag (Myc-sense and Myc-antisense) (Table S1) were annealed and inserted into the *Nhe*I-*Hind*III-pretreated pcDNA3.1(+)-Hyg (Invitrogen). The construction of pSK-eIF4GI was as described previously [23]. To generate the plasmid expressing Flag-tagged or myc-tagged eIF4GI, pSK-eIF4GI was treated with *Not*I, *Hind*III and *Klenow* sequentially, and inserted into the *Bam*HI-*Klenow*-treated pcDNA3.1-Flag or pcDNA3.1-myc. To prepare the eIF4GI deletion mutants, the corresponding fragments (Tables S2 and S3) were synthesized by PCR using the *Pyrobest™* DNA polymerase (TaKaRa) and inserted into the *Hind*III-*Ksp*I-pretreated pEGFP-C1 (Clontech). Subsequently, the pEGFP-C1-eIF4GI variants were treated with *Hind*III, *Ksp*I and T4 DNA polymerase (T4Pol) sequentially, and cloned into the *Bam*HI-*Klenow*-treated pcDNA3.1-Flag or pcDNA3.1-myc.

To obtain the plasmids expressing myc-tagged Ago1 or Ago2, pIRESneo-Flag/HA-Ago1 or pIRESneo-Flag/HA-Ago2 (kindly provided by Dr. Thomas Tuschl, Rockefeller University, USA) were treated with *Not*I, Mung Bean nuclease (*MB*), and *Bam*HI, and then inserted into the *Kpn*I-*T4Pol*-*Bam*HI-treated pcDNA3.1-myc. To generate the plasmids expressing the full-length Ago2 and its variants fused with GFP, the corresponding fragments were synthesized by PCR (Tables S4 and S5) and inserted into the *Hind*III-*Eco*RI-treated pEGFP-C1. To generate the plasmids expressing the myc-tagged Ago2 deletion mutants, the DNAs encoding the GFP-fused Ago2 variants were treated with *Hind*III, *Ksp*I and *T4Pol*, and then inserted into the *Bam*HI-*Klenow*-treated pcDNA3.1-myc. To construct the plasmids expressing the Flag-tagged full-length Ago1 or Ago2, the DNAs encoding myc-tagged Ago1 or Ago2 were treated with *Hind*III and *Not*I, and then inserted into the *Hind*III-*Not*I-treated pcDNA3.1-Flag. To make pcDNA3-λN-Flag, pcDNA3-Flag (kindly provided by Dr. Didier Poncet), oligonucleotides for λN peptides (λN-sense and λN-antisense) (Table S1) were annealed and inserted into the *Nhe*I site of pcDNA3-Flag. To construct the plasmids expressing the λN-Flag-tagged Ago2 series, the DNA fragments were PCR-amplified (Tables S6 and S7) and inserted into the *Not*I-*Bam*HI site of pcDNA3-λN-Flag.

To obtain a vector encoding the TNRC6C gene (accession No. NM_001142640), the nested PCR was performed using a human cDNA library (Clontech) and the appropriate primers (Table S8), and the amplified DNA was inserted into the *Hind*III-*Not*I site of pcDNA3.1-Flag. To obtain a vector encoding the eIF4E gene (accession No. NM_001968), PCR was performed using the human cDNA library and the appropriate primers (Table S8), and the amplified DNA was inserted into the *Xba*I-*Eco*RI site of pSK(−). To generate pEGFP-C1-eIF4E, the *Xba*I-*Klenow*-*Eco*RI treated fragment of pSK(−)-eIF4E corresponding to the open reading frame of the eIF4E was inserted into pEGFP-C1 that had been treated with *Hind*III, *Klenow* and *Eco*RI. To generate the plasmid expressing the Flag-tagged eIF4E, PCR was performed using the human cDNA library and the appropriate primers (Table S8), and the amplified DNA was inserted into the *Hind*III-*Not*I site of pcDNA3.1-Flag. To obtain the plasmids encoding eIF3c (the p110 subunit of the eIF3 complex; accession No. NM_003752) and PABP (accession No. NM_002568), the genes were amplified by PCR (Table S8) and inserted into the *Hind*III-*Not*I and *Kpn*I-*Bam*HI sites of pcDNA3.1-Flag, respectively.

To construct pcDNA3.1-FL, pGL3-Enhancer (Promega) was treated with *Hind*III, *Xba*I and *Klenow*, and the resulting fragment was inserted into pcDNA3.1(+)-Hyg that had been pre-treated with *Hind*III and *Klenow*. The plasmids expressing the FL mRNA with three or six imperfect binding sites for si-CXCR4 at the 3′-UTR were constructed as previously described [24]. Briefly, oligonucleotides containing three tandem repeats of an imperfect binding site for si-CXCR4 (CXCR4-3×Bulge-sense and CXCR4-3×Bulge-antisense, see Table S1) were annealed and inserted into the *Bgl*II-*Eco*RV site of pcDNA3.1-FL to construct pcDNA3.1-FL-3×Bulge. To generate pcDNA3.1-FL-6×Bulge, the *Kpn*I-*T4Pol*-*Not*I treated fragment of pcDNA3.1-FL-3×Bulge was inserted into the *Eco*RV-*Not*I site of pcDNA3.1-FL-3×Bulge. For the *in vitro* preparation of the ∼350-nt-long RNAs used in the cap-crosslinking assays, pcDNA3.1-6×Bulge was constructed by inserting the *Kpn*I-*Not*I fragment of pcDNA3.1-FL-6×Bulge into the *Kpn*I-*Not*I site of pcDNA3.1(+)-Hyg. For plasmids expressing the poly(A)-tailed mRNAs, pcDNA3.1-FL-6×Bulge was treated with *Not*I followed by *Klenow*. Then, the *Xho*I-*Xba*I-*Klenow*-treated (A)_120_ fragments from pSK-(A)_120_ were inserted at that region. All of the above-described plasmids were verified by sequencing.

Finally, pcDNA3.1-Flag-Dicer was a gift from Dr. Patrick Provost (Université Laval, Canada); pcDNA3.1-Flag-eIF4AI was kindly provided by Dr. Yongjun Dang (Johns Hopkins University); and pRL-CMV was purchased from Promega.

### Antibodies

The rabbit anti-eIF4GI and anti-eIF4GII were as described previously [23]. The rat monoclonal Ago2-specific antibody (11A9) was kindly provided by Dr. Gunter Meister (Max Planck Institute of Biochemistry). The mouse anti-Myc (9E10) was kindly provided by Dr. Sung Ho Ryu (POSTECH, Korea). The mouse and rabbit anti-Flag antibodies were purchased from Sigma. The rabbit anti-eIF4E antibody was purchased from Abcam. The rabbit anti-Dicer, anti-eIF3c and anti-Myc, the goat anti-eIF4A, and the mouse anti-eIF4E, anti-HuR, anti-GW182 (4B6) and anti-PABP (10E10) antibodies were all purchased from Santa Cruz. The mouse anti-GAPDH antibody was purchased from AbD SEROTEC. In each experiment, all of immunoblottings were done using the same membrane by stripping and reprobing methods.

### Synthetic siRNAs

All of siRNAs were purchased from Bioneer. The sequences of the siRNAs against eIF4GI and eIF4GII were designed using the Dharmacon siDESIGN® center (http://www.dharmacon.com/DesignCenter/DesignCenterPage.aspx). The sense strands of the siRNAs were as follows: si-Control (also called miControl in the miRNA-mediated translational repression assay), 5′-CCU ACG CCA CCA AUU UCG UTT-3′; si-eIF4GI, 5′-UGA GAA AGG AGG AGA GGA ATT-3′; si-eIF4GII, 5′-CCA CGC CUG UAG AGU UUG ATT-3′; si-Ago2, 5′-GCA CGG AAG UCC AUC UGA ATT-3′ [25]; si-PABP, 5′-AAG GUG GUU UGU GAU GAA AAU TT-3′ [26]; and si-CXCR4 (denoted as miCXCR4), 5′-GUU UUC ACU CCA GCU AAC ACA-3′ [24].

### Cell culture, transfection and luciferase assay

HeLa or 293FT (Invitrogen) cells were cultivated in DMEM (Gibco BRL) supplemented with 10% FBS (Clontech). Transfections were carried out using Lipofectamine PLUS for the introduction of DNAs into cells, Lipofectamine 2000 for the co-delivery of both DNAs (or RNAs) and siRNAs, and Oligofectamine for siRNAs alone (all from Invitrogen). For the dual luciferase reporter assay, synthetic siRNAs (100 nM) were first transfected into ∼40% confluent HeLa cells grown on a 24-well plate. At 24 h post-transfection, FL-expressing reporter species (50 ng of pcDNA3.1-FL or pcDNA3.1-FL-6×Bulge for the DNA transfection; 100 ng of FL-expressing mRNAs for the RNA transfection) and RL-expressing controls (20 ng of pRL-CMV for the DNA transfection, Promega; 20 ng of RL-expressing mRNAs for the RNA transfection) were co-transfected with miControl or miCXCR4 (5 nM each). After incubation for 24 h (19 h for the RNA transfection), the cells were rinsed with cold PBS (pH 7.4) and lysed, and luciferase assays were performed according to the manufacturer's recommendations (Promega). The translational efficiencies of the reporter mRNAs were normalized by dividing the FL activity by the RL activity; this compensated for the general translational inhibition induced by the knock-down of translation factors, as well as for differences in transfection efficiency. To see a change in the miRNA-mediated translational repression (called the fold induction), if not mentioned, the FL/RL activities in miCXCR4-treated cells were normalized with respect to those from miControl-treated cells. The ratios were then relatively shown by setting the values from miControl/si-Control-treated cells to 1.

### Immunoprecipitation and cap-pulldown assay

293FT cells were cultivated on plates, washed with cold PBS (pH 7.4), harvested using 300–700 µl of ice-cold lysis buffer [0.1% NP-40, 40 mM HEPES-KOH (pH 7.5), 100 mM KCl, 1 mM EDTA, 10 mM β-glycerophosphate, 10 mM NaF, 2 mM Na_3_VO_4_, and 1 mM PMSF], sonicated on ice, and centrifuged to yield whole-cell extracts (WCEs). For immunoprecipitation, WCEs were incubated with 10 µl of anti-Flag M2 affinity gel (Flag-resin; Sigma) at 4°C for 2 h with constant rotation. The beads were collected and washed four times with the same buffer. The bead-bound proteins were resolved by SDS-PAGE and analyzed by Western blotting. For the cap-pulldown assays, 10–20 µl of m^7^GTP Sepharose 4B (cap-resin; GE Healthcare) was used in place of the Flag-resin. If necessary, the equal amount of Glutathione Sepharose 4B (control-resin; GE Healthcare) was used as a negative control. In some cases, m^7^G(5′)ppp(3′)G or G(5′)ppp(3′)G (200 µM each; NEB) was included as a competitor. When RNase A treatment was desired, WCEs were pre-incubated with 10 µg/ml RNase A (Sigma) on ice for 15 min and pre-cleared with protein G agarose beads at 4°C for 1 h prior to the samples being used for immunoprecipitation or the cap-pulldown assay. Unless otherwise indicated, 1 mg WCEs were used in the binding assays and 40 µg WCEs were loaded on the SDS-PAGE gels as an input control.

### *In vitro* transcription for the reporter mRNAs

The plasmids pcDNA3.1-FL-6×Bulge series with *Not*I, pcDNA3.1-FL-6×Bulge-(A)_120_ treated with *Nsi*I followed by T4 DNA polymerase, and pRL-CMV treated with *Xba*I were used for the templates for the *in vitro* transcription mediated by T7 RNA polymerase to generate various kinds of luciferase-expressing mRNAs. To produce capped mRNAs, m^7^G(5′)ppp(3′)G or A(5′)ppp(3′)G cap analogs were included during the reaction.

### Cap photo-crosslinking assay

To generate ∼350-nt-long RNAs, we used *Not*I-treated pcDNA3.1-6×Bulge as the template for *in vitro* transcription by T7 RNA polymerase. The resulting RNAs were subjected to an *in vitro* capping reaction with [α^32^P]-GTP using the ScriptCap™ m^7^G Capping System (Epicentre) according to the manufacturer's recommendations. The 5′ cap-labeled RNAs were purified previously described [27]. The radioactivity was measured using a Liquid Scintillation Analyzer (TRI-CARB 2900TR, Packard BioScience Company).

The cap photo-crosslinking experiments were similar to the *in vitro* RNAi assay described by Lee et al. [28]. Briefly, Flag-tagged proteins were pulled down using the Flag-resin. The protein-bound beads were washed twice with the RNAi buffer [30 mM HEPES-KOH (pH 7.5), 40 mM KOAc, 5 mM MgOAc, 5 mM DTT and 5 ng/µl of yeast tRNAs], and the cap-labeled RNAs (∼200,000 cpm) were incubated with the beads in the presence of 2 U/µl RNasin (Solgent) in the absence or presence of cap analog (200 µM) at 37°C for 90 min and then photo-crosslinked with 254 nm of wavelength (UV_254_) for 30 min in ice using an UV irradiator (UVP). The unbound RNAs were digested by 1 µg/µl RNase A and 10 U/µl RNase T1 (Roche) at 37°C for 30 min. The beads were washed once with the lysis buffer and the protein-RNA complexes were resolved by SDS-PAGE and visualized by autoradiography.

### Quantitative RT-PCR analysis and Northern blotting of mRNA or miRNA

Purification of total RNAs was performed using Qiazol (Qiagen) as manufacturer's recommendations. Quantitative RT-PCR analysis [29] and Northern blotting of mRNA [30] were performed as previously described. The cap-pulldown assay was followed by miRNA Northern blotting, similar to the previously described protocol for immunoprecipitation followed by miRNA Northern blotting [31]. Oligonucleotides against the let-7a (5′-AAC TAT ACA ACC TAC TAC CTC A-3′), miR-16 (5′-CGC CAA TAT TTA CGT GCT GCT A-3′), miR-21 (5′-GTC AAC ATC AGT CTG ATA AGC TA-3′) miRNAs or U6 (5′-GCA GGG GCC ATG CTA ATC TTC TCT GTA TCG-3′) snRNAs, all of which had been 5′-end-labeled with ^32^P using T4 PNK1 (TaKaRa), were used as miRNA Northern probes.

## Results

### Ago2 associates with the cap-binding complex

**Figure 1:**
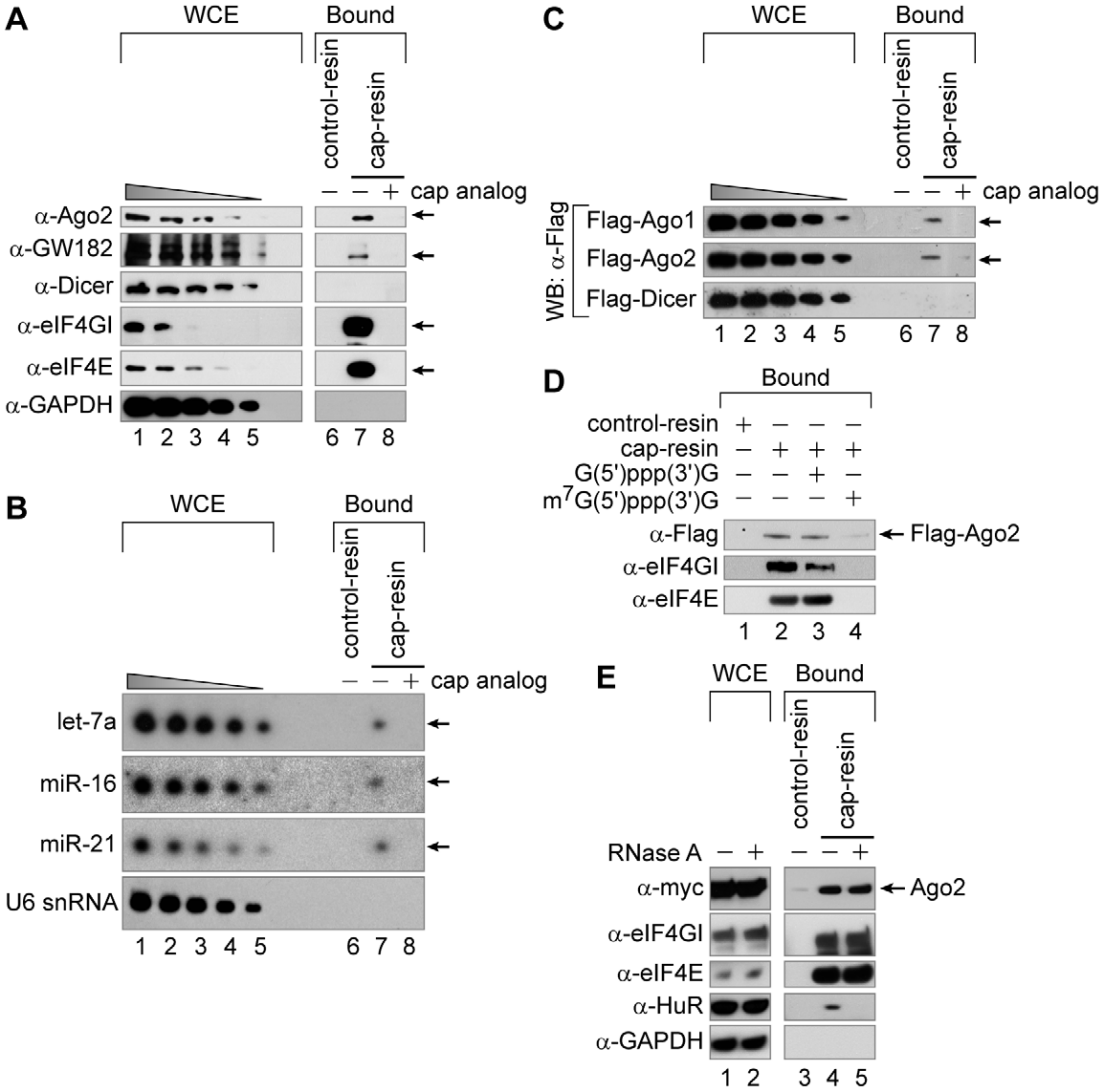
Human Ago associates with the cap-binding complex. (*A*) The cap-association of endogenous Ago2 proteins from HeLa cells were examined by a cap-pulldown assay, using 2 mg of whole-cell extracts (WCEs) for incubation with either control-resin (lane 6) or cap-resin in the presence (lane 8) or absence (lane 7) of the cap analog. (*B*) The cap-association of miRNAs. WCEs from HeLa cells (2 mg) were subjected to a cap-pulldown assay, and the cap-associated RNAs were extracted and subjected to UREA-PAGE followed by Northern blotting using radiolabeled probes against the indicated miRNAs. For comparison, various amounts equal to 4.4–0.8% of the total RNAs contained in WCEs used for the cap-pulldown assays (∼10–2 µg each) were loaded in lanes 1–5. (*C*) The cap-associations of ectopically expressed proteins were monitored as in panel *A*, except for using 2 mg of WCEs from 293FT cells transfected with plasmids expressing Flag-tagged Ago1, Ago2 or Dicer. (*D*) Cap-pulldown assays were done using 2 mg of WCEs from 293FT cells expressing Flag-Ago2 with 200 µM of G(5′)ppp(3′)G (lane 3) or m^7^G(5′)ppp(3′)G (lane 4). (*E*) The RNA-independent cap-association of Ago2. 2 mg of WCEs from 293FT cells ectopically expressing myc-tagged Ago2 were treated with (lanes 2 and 5) or without (lanes 1, 3 and 4) RNase A and subjected to cap-pulldown assays. In panels *A* and *C*, various amounts corresponding to 2–0.4% of WCEs used in the pulldown assay were loaded in lanes 1–5 for comparison. In panels *A*, *C*, *D* and *E*, Western blot analyses were performed using the indicated antibodies.

It was reported that miRNA can inhibit the protein synthesis at the initiation step of translation [32], proposing that the RNA-induced silencing complex (RISC) or the miRNA-containing ribonucleotide protein complex (miRNP) could associate with the cap-binding complex (e.g. eIF4F complex) to facilitate the miRNA-mediated gene silencing. Therefore, we empirically tested whether a component of the RISC (or the miRNP) such as Ago2 could associate with the cap-binding complex. HeLa cell extracts were subjected to a cap-pulldown assay with the cap-resin in the absence or presence of the cap analog (Figure 1A, lanes 7 and 8, respectively) or the control-resin (Figure 1A, lane 6). We found that Ago2 precipitated with the cap-resin, and their association decreased with the addition of cap analog (Figure 1A). The well-known Ago-interacting protein GW182 also associated, albeit weakly, with the cap-resin, whereas Dicer, an essential protein for the pre-miRNA processing, did not (Figure 1A). As expected, the components of the cap-binding complex (eIF4E and eIF4GI) specifically precipitated with the cap-resin, whereas the negative control protein (GAPDH) did not (Figure 1A). Similarly, miRNAs (let-7a, miR-16 and miR-21), but not the U6 snRNA (a component of snRNP), were detected in cap-pulldown assays followed by Northern blotting (Figure 1B, lane 7), suggesting that the miRNP associates with the cap-binding complex (hereafter, the silencing complex, which contains Ago, GW182, etc., but excludes Dicer, is arbitrarily denoted as the miRNP). Ectopically expressed Ago1 and Ago2 also associated with the cap-resin (Figure 1C, lane 7), while ectopically expressed Dicer did not, consistent with the results in Figure 1A. The association of Ago2 with the cap-resin disappeared by inclusion of m^7^G(5′)ppp(3′)G, but not G(5′)ppp(3′)G, during the binding experiment, indicating the specific association of Ago2 with the cap-resin (Figure 1D, compare lane 3 with lane 4). We then used RNase A-treated or -untreated cell extracts expressing myc-tagged Ago2 to investigate whether the Ago2-cap association is mediated by RNAs. As shown in Figure 1E, pre-treatment of the cells with RNase A did not interfere with the binding of eIF4GI and eIF4E to the cap-resin, but it did abrogate the cap-association of an RNA-binding protein HuR. Most notably, the Ago2-cap association was not altered by RNase treatment, indicating that it is independent of RNAs (Figure 1E, lanes 4 and 5). Collectively, these data indicate that the miRNP complex specifically associates with the cap-binding complex through protein-protein interaction(s) in an RNA-independent manner.

### eIF4GI facilitates the Ago2-cap association

**Figure 2:**
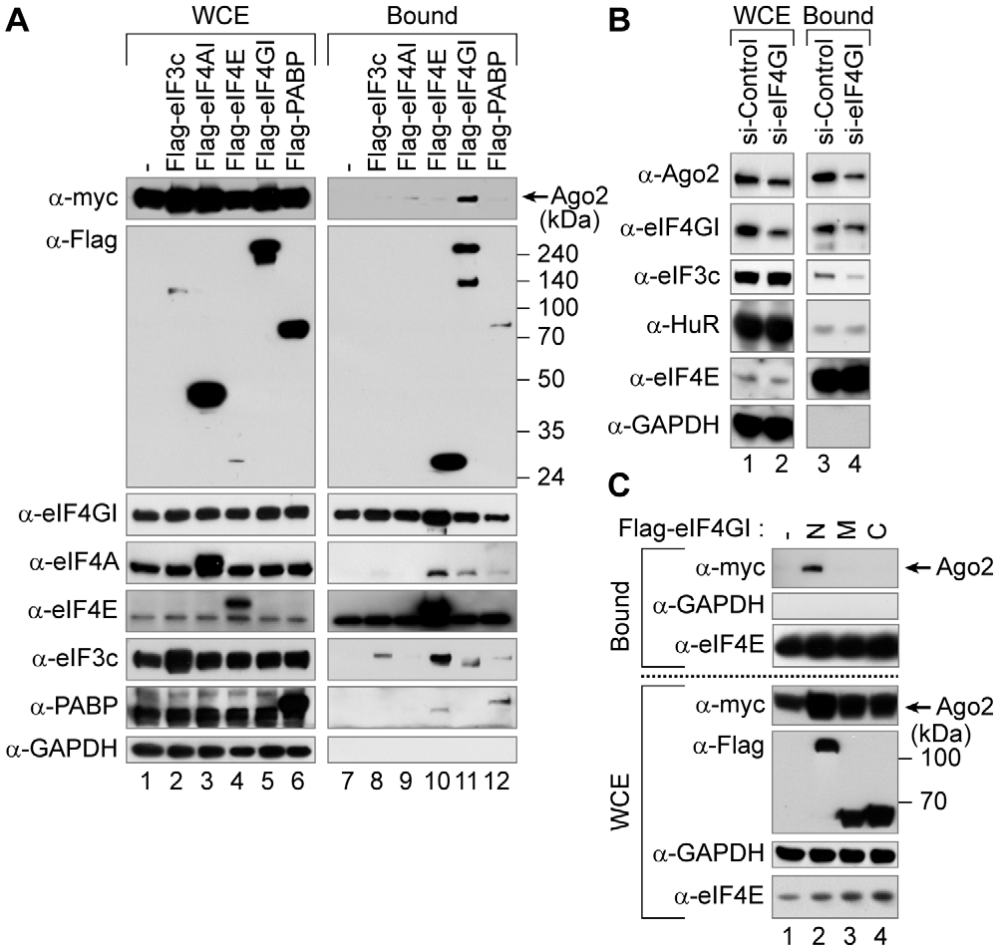
eIF4GI mediates the Ago2-cap association. (*A*) Identification of a translation initiation factor capable of augmenting the Ago2-cap association. WCEs from 293FT cells co-transfected with plasmids expressing myc-Ago2 and Flag-tagged translation factors (eIF3c, eIF4AI, eIF4E, eIF4GI and PABP) were subjected to cap-pulldown assays. The expression levels of the transfected genes (lanes 1–6), their cap-associations (lanes 7–12), and various proteins from WCEs or from the resin-bound fractions were monitored by Western blotting with the indicated antibodies. (*B*) The eIF4GI-dependent cap-association of Ago2. WCE from HeLa cells transfected with si-Control or si-eIF4GI were applied to cap-pulldown assays. The knock-down efficiency of siRNAs and the resin-bound proteins were monitored using antibodies described. (*C*) Determination of a domain in eIF4GI responsible for augmenting the Ago2-cap association. WCEs from 293FT cells transiently expressing myc-Ago2 and Flag-tagged fragments encoding the N-terminal, middle or C-terminal regions of eIF4GI (depicted in Figure 5A) were subjected to cap-pulldown assays. The resin-bound Ago2 (upper panel), the ectopically expressed Ago2 and eIF4GI fragments (lower panel), GAPDH and eIF4E proteins were detected using the indicated antibodies.

In an attempt to identify factor(s) which might facilitate the Ago2-cap association, we focused particularly on the translation initiation factors such as eIF3c, which is a component of eIF3 complex, poly(A)-binding protein (PABP), and the components of eIF4F complex like eIF4E, eIF4AI and eIF4GI which associate with the 5′ cap structure to promote the assembly of the ribosome onto the mRNA. 293FT cells were co-transfected with plasmids expressing myc-tagged Ago2 and Flag-tagged translation initiation factors (eIF3c, eIF4AI, eIF4E, eIF4GI and PABP), and cell extracts were subjected to cap-pulldown assays. To examine an increase in the Ago2-cap association by an initiation factor easily, we used smaller amount (1 mg) of whole cell extracts (WCE) in these experiments compared with those used in Figure 1 (2 mg). Our results revealed that the association of Ago2 with the cap-resin was enhanced by the ectopic expression of eIF4GI (Figure 2A, lane 11). However, the ectopic expressions of eIF4AI, eIF3c, PABP and eIF4E did not increase the Ago2-cap association though their amounts of expressions were similar to or greater than those of endogenous ones (Figure 2A). The augmented Ago2-cap association by Flag-tagged eIF4GI was not likely due to the increased level of myc-tagged Ago2 because the ectopic expressions of eIF3c and eIF4AI, which resulted in the higher level of myc-tagged Ago2 (Figure 2A, compare lanes 2, 3 and 5 with lane 1), did not affect the Ago2-cap association (Figure 2A, compare lanes 8 and 9 with lane 11). Conversely, the knock-down of endogenous eIF4GI by si-eIF4GI decreased the Ago2-cap association as well as the eIF3c-cap association which is mediated by the eIF4GI-eIF3 interaction via the middle domain of eIF4GI [13] (Figure 2B, lanes 3 and 4 on the panels α-Ago2 and α-eIF3c), but it did not affect the HuR-cap association which occurs in an eIF4GI-independent manner (Figure 2B, lanes 3 and 4 in the α-HuR portion), implying that Ago2 can associate with the cap-binding complex with the help of eIF4GI. We further investigated which part of eIF4GI is responsible for assisting the Ago2-cap association (Figures 2C and 5A). Ectopic expression of the N-terminal part of eIF4GI (aa 42–622) facilitated the Ago2-cap association (Figure 2C, lane 2). In contrast, the middle (aa 623–1090) and C-terminal (aa 1091–1600) domains did not (Figure 2C, lanes 3 and 4) although all of the eIF4GI series increased the amount of myc-tagged Ago2 than the control experiment (Figure 2C, compare lanes 2–4 with lane 1 in the α-myc portion of the lower panel). These data suggest that eIF4GI promotes the Ago2-cap association through its N-terminus.

### eIF4GI participates in the miRNA-mediated translational repression

**Figure 3:**
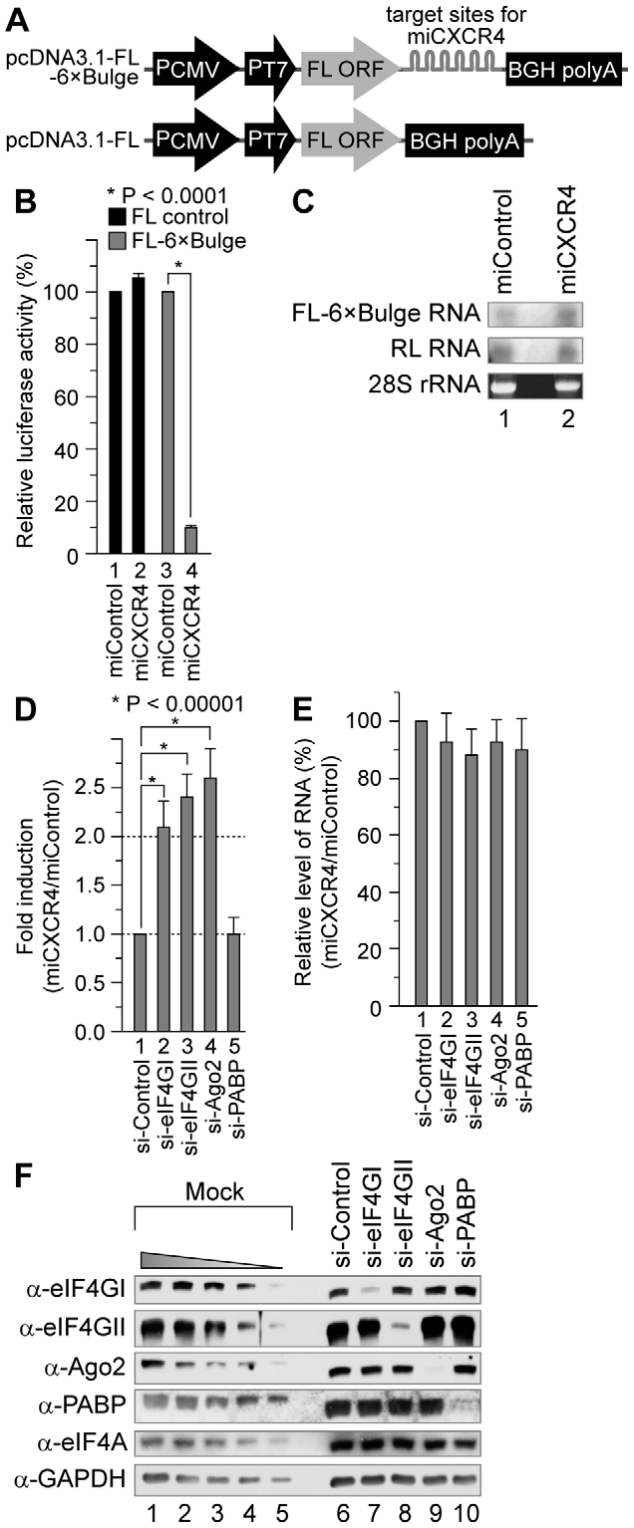
eIF4GI participates in the miRNA-mediated gene silencing. (*A*) Schematic diagrams of the reporter constructs for the investigation of the miRNA-mediated gene silencing. pcDNA3.1-FL-6×Bulge expresses a FL mRNA containing six imperfect repeats of the miCXCR4-binding site. pcDNA3.1-FL expresses a FL mRNA lacking the miCXCR4-binding site. Abbreviations: P_CMV_, the CMV promoter; P_T7_, the T7 promoter; FL, firefly luciferase. (*B*) HeLa cells cultivated on a 24-well plate were transfected with the reporter plasmids in the presence of miControl or miCXCR4. After 24 h of incubation, the luciferase activities were measured by a dual luciferase assay. The activity ratios of FL to RL (FL/RL) were calculated, and the relative values were determined by setting the value from miControl-treated cells to 100%. (*C*) Total RNAs from the cells described in panel *B* were extracted, and the levels of reporter mRNAs were analyzed by Northern blotting. The EtBr-staining of 28S rRNAs was assessed as a control for total RNA levels. (*D*) De-repression of the miRNA-mediated gene silencing by siRNAs against translation initiation factors. The overall experimental procedures and analyses were described in the Materials and Methods. (*E*) The relative amounts of the reporter mRNAs in panel *D* were measured by quantitative RT-PCR analysis. The ratios of FL/RL mRNAs obtained from miCXCR4-treated cells were first normalized by that of miControl-treated cells and then relatively presented by setting the value from miControl/si-Control-treated cells to 100% (lane 1). (*F*) The knock-down efficiency of each siRNA was examined by Western blotting using the indicated antibodies. For comparison, WCEs from mock-transfected HeLa cells (100–0%; denoted as Mock) were loaded in lanes 1–6. All experiments were performed in triplicate. In panels *B* and *D*, the P-values are described (Student *t*-test). Standard deviations are indicated by bars.

Since eIF4GI facilitates the Ago2-cap association (Figure 2), we investigated whether it participates in the miRNA-mediated translational repression. For this, we used a reporter plasmid (pcDNA3.1-FL-6×Bulge) capable of generating an mRNA whose translation is repressed by the exogenous si-CXCR4 in human cells. This system was previously shown to mimic the translational repression by miRNAs [24]. Reporter gene expression from an mRNA containing the target sites for si-CXCR4 (FL-6×Bulge) was repressed by about 90% in cells transfected with si-CXCR4 (hereafter, this small RNA is referred to as ‘miCXCR4,’ since it functions like a miRNA) (Figure 3B, lane 4). In contrast, the reporter gene expression from an mRNA lacking the miCXCR4 target site (FL control) was not affected by the presence of miControl or miCXCR4 (Figure 3B, lanes 1 and 2). Northern blot analyses showed that the levels of FL-6×Bulge mRNAs did not decrease by miCXCR4 (Figure 3C). These data indicate that miCXCR4 specifically represses the gene expression at the translational level, confirming its suitability for analyzing the roles of cellular proteins in the miRNA-mediated translational regulation as reported previously [6], [24], [25].

Next, we knocked down translation factors, and examined their effects on the miRNA-mediated gene silencing using the reporter system described above (Figures 3D–F). We found that a siRNA against Ago2 de-repressed the protein synthesis of the miCXCR4-repressed mRNAs by more than 2.5-fold as expected (Figure 3D, lane 4). Importantly, siRNAs against eIF4GI and eIF4GII also de-repressed the protein synthesis of the miCXCR4-repressed mRNAs by about 2 fold (Figure 3D, lanes 2 and 3). On the other hand, a siRNA against PABP showed no effect on the miRNA-mediated gene silencing in this system (Figure 3D, lane 5). There was no apparent change in the relative levels of the reporter mRNAs following siRNA treatment (Figure 3E). The knock-down of the siRNA-targeted proteins was confirmed by Western blotting (Figure 3F).

**Figure 4:**
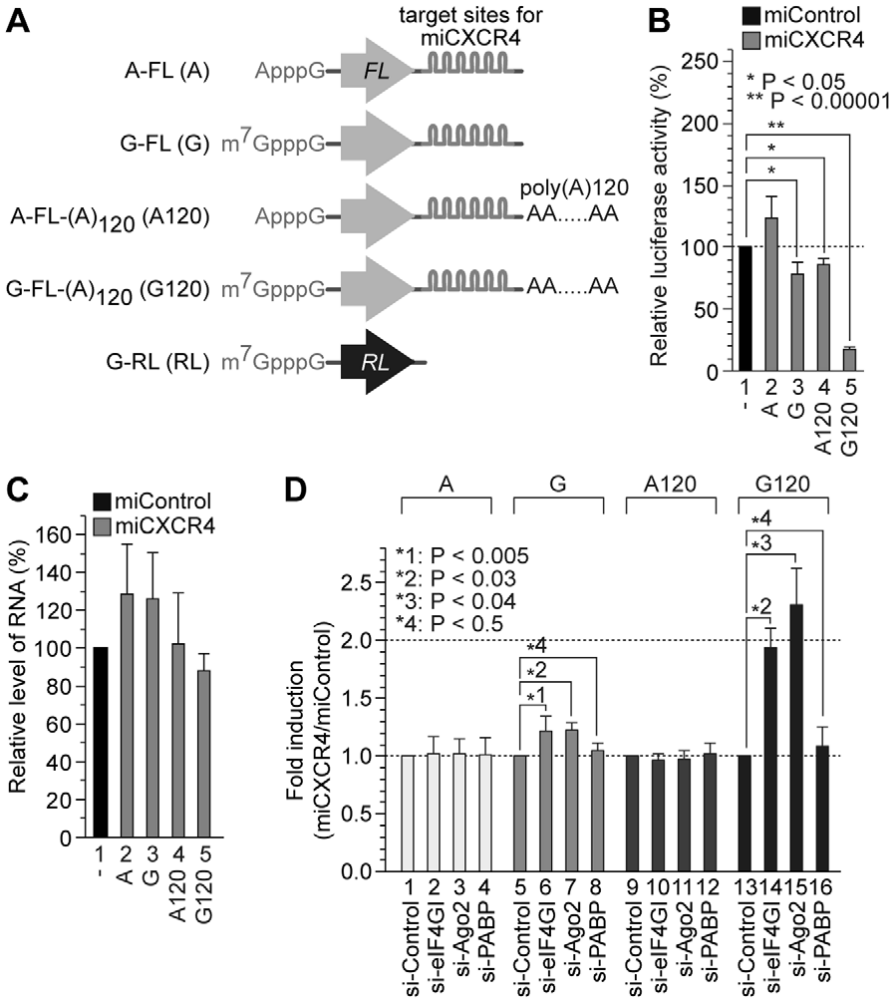
eIF4GI plays a role in the miRNA-mediated gene silencing of the poly(A)-tailed m^7^G-capped mRNA. (*A*) Schematic diagrams of the reporter mRNAs used here. The abbreviations of each reporter are depicted in parentheses at the left side. (*B*) The effect of the 5′ cap structure and the poly(A) tail on the miRNA-mediated translational repression. m^7^G-capped/A-capped and/or poly(A)-tailed/nonadenylated FL mRNAs were co-transfected with the nonadenylated m^7^G-capped RL mRNAs in the presence of miControl or miCXCR4 into HeLa cells. After 19 h of incubation, the luciferase activities were measured to obtain the FL/RL activity ratios. (*C*) Total RNAs from the cells in panel B were extracted, and the relative levels of the mRNAs were analyzed by quantitative RT-PCR analysis. In panels *B* and *C*, the values from miControl-treated cells in all experiments were represented as 100% (lane 1). (*D*) De-repression of the miRNA-mediated gene silencing by siRNAs against translation factors using the RNA reporter system. The overall procedures for experiments and analyses were described in the Materials and Methods. All experiments were performed in triplicate. In panels *B* and *D*, the P-values are described (Student *t*-test). Standard deviations are indicated by bars.

It is known that the 5′ cap-dependent mRNAs with the poly(A) tail can be translationally repressed by miRNAs but the IRES-driven translation cannot [6], [7], [10], [33]. Indeed, translation of the mRNA containing both the 5′ cap structure and the 3′ poly(A) tail was repressed up to 80% in the presence of miCXCR4 (Figure 4B, lane 5). On the other hand, translation of mRNAs lacking either the 5′ cap structure or the 3′ poly(A) tail (Figure 4B, lanes 4 and 3, respectively) was repressed only slightly (less than 20%). Taken together, these data reconfirm the previous reports suggesting that both 5′ cap structure and 3′ poly(A) tail are needed for the efficient translational repression by miRNAs. These results may suggest that a functional communication between the 5′ and 3′ ends of mRNA, i.e., a crosstalk between the eIF4F complex assembled at the 5′ cap structure and the PABP-containing protein complex congregated onto the 3′ poly(A) tail, is required for translational repression by miRNAs.

In order to know putative roles of eIF4GI in the miRNA-mediated repression of the translation initiation where the 5′ cap structure and the 3′ poly(A) tail are involved, we performed miRNA-mediated translation repression assays in HeLa cells using various reporter mRNAs, which are described in Figure 4A, in the presence or the absence of miCXCR4 (Figure 4D). The miRNA-mediated translational repression of the m^7^G-capped and poly(A)-tailed mRNAs was de-repressed about 2 folds by knock-down of either eIF4GI or Ago2 (Figure 4D, compare lanes 14 and 15 with lane 13). A slight de-repression (about 20%) of the miRNA-mediated translational repression of the m^7^G-capped mRNAs lacking the poly(A) tail was observed from the cells transfected with either si-eIF4GI or si-Ago2 (Figure 4D, compare lanes 6–7 with lane 5). On the other hand, de-repression was not observed from the A-capped reporters with (lanes 9–12 in Figure 4D) or without poly(A) tail (lanes 1–4 in Figure 4D). The knock-down of PABP did not show apparent de-repression effect on the miRNA-mediated silencing (Figure 4D, lanes 4, 8, 12 and 16), similarly to the results shown in Figure 3D. A plausible reason for this effect will be described in the Discussion section. In conclusion, these data imply that both eIF4GI participates in the miRNA-mediated repression of the mRNA translation which is synergistically activated by the 5′ cap structure and the 3′ poly(A) tail.

### eIF4GI associates with Ago2

**Figure 5:**
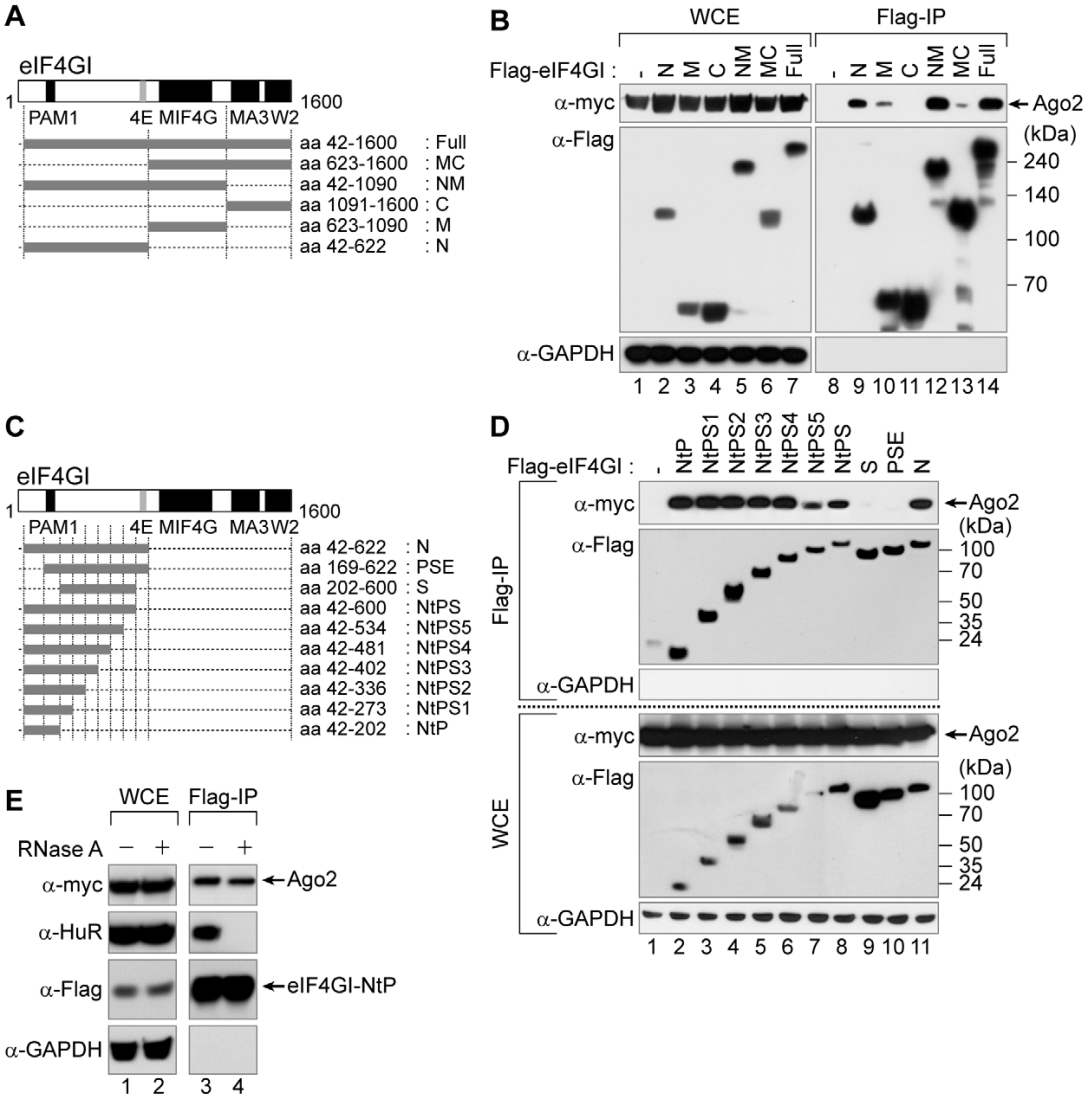
eIF4GI associates with Ago2. (*A*) Schematic diagram of human eIF4GI. ‘4E’ means ‘the eIF4E-binding motif’. Plasmids were constructed for expression of various Flag-tagged eIF4GI fragments in human cells. (*B*) The N-terminal and middle domains of eIF4GI participate in the eIF4GI-Ago2 association. Plasmids expressing Flag-tagged eIF4GI variants and myc-tagged full-length Ago2 were co-transfected in 293FT cells, and their associations were examined by Flag immunoprecipitation (Flag-IP) with the Flag-resin. The levels of Ago2 and the eIF4GI mutants (left panel) and the amount of co-precipitated Ago2 (right panel) were monitored by Western blotting using the indicated antibodies. (*C*) Schematic diagram of the N-terminal constructs of eIF4GI for fine mapping of the region required for the association with Ago2. (*D*) Determination of the Ago2-associated region in eIF4GI. WCEs from 293FT cells expressing myc-tagged full-length Ago2 and N-terminal variants of eIF4GI serially deleted from the C- or N-termini were subjected to Flag-IP. The expressions of Ago2 and the eIF4GI derivatives (lower panel) and the amount of precipitated Ago2 (upper panel) were examined using the indicated antibodies. (*E*) RNA-independent association of Ago2 with eIF4GI (aa 42–202). WCEs from 293FT cells expressing myc-tagged Ago2 and Flag-tagged eIF4GI-NtP were treated with (lanes 2 and 4) and without (lanes 1 and 3) RNase A and subjected to Flag-IP.

Since both eIF4GI and Ago2 are required for the translational repression by miRNAs (Figures 3 and 4), we examined whether they could form a complex in cells. We found that Ago2 co-precipitated with the full-length eIF4GI (Figure 5B, lane 14). In co-immunoprecipitation experiments with fragments of eIF4GI, Ago2 co-precipitated with the N-terminal domain (aa 42–622) and rather weakly with the middle domain (aa 623–1090) (Figure 5A; Figure 5B, compare lane 9 with lane 10). The NM domain (aa 42–1090) of eIF4GI associated with Ago2 better than the N domain of eIF4GI (Figure 5A; Figure 5B, compare lane 9 with lane 12). In contrast, the C-terminal region of eIF4GI did not associate with Ago2 (Figure 5B, lane 11). These results indicate that the N-terminal region of eIF4GI mainly associates with Ago2, and the middle domain augments this association.

To further define a region in eIF4GI responsible for the association with Ago2, we focused on the N-terminal portion because it had augmented the cap-association of Ago2 in our earlier experiments (Figure 2C). As shown in Figure 5D, further deletion from the N-terminal end of eIF4GI to aa 168 (PSE) or more (S) resulted in loss of the Ago2-associating activity (Figure 5D, compare lanes 9–10 with lane 11). This indicates that the N-terminal border of eIF4G required for Ago2-association resides between aa 42–168 of eIF4GI. Serial deletions from the C-terminus of the eIF4GI-N domain showed that deletion up to aa 202 (NtP) maintained the association with Ago2 (Figure 5D, lanes 2–8). In conclusion, aa 42–202 of eIF4GI is sufficient for the eIF4GI-Ago2 association. This minimal region for the eIF4GI-Ago2 association also had an RNA-independency in the cells (Figure 5E, compare lane 3 with lane 4).

Next, we determined the minimal region required for eIF4GI to augment the cap-association of Ago2. The N-terminus (aa 42–622) of eIF4GI facilitated the Ago2-cap association as expected but either NtP (aa 42–202) or NtPS (aa 42–600) did not (data not shown), although they associated with Ago2 (Figure 5D, compare lanes 2 and 8 with lane 11). This indicates that the eIF4GI-Ago2 association is not sufficient to mediate the Ago2-cap association. Instead, a region spanning aa 601–622 of eIF4GI, which includes the eIF4E-binding motif, is required for this activity, suggesting that eIF4E may participate in the Ago2-cap association although we observed no change in the Ago2-cap association by the overexpression of eIF4E (Figure 2A, lane 10).

### The intact Ago2 associate with the cap-binding complex

**Figure 6:**
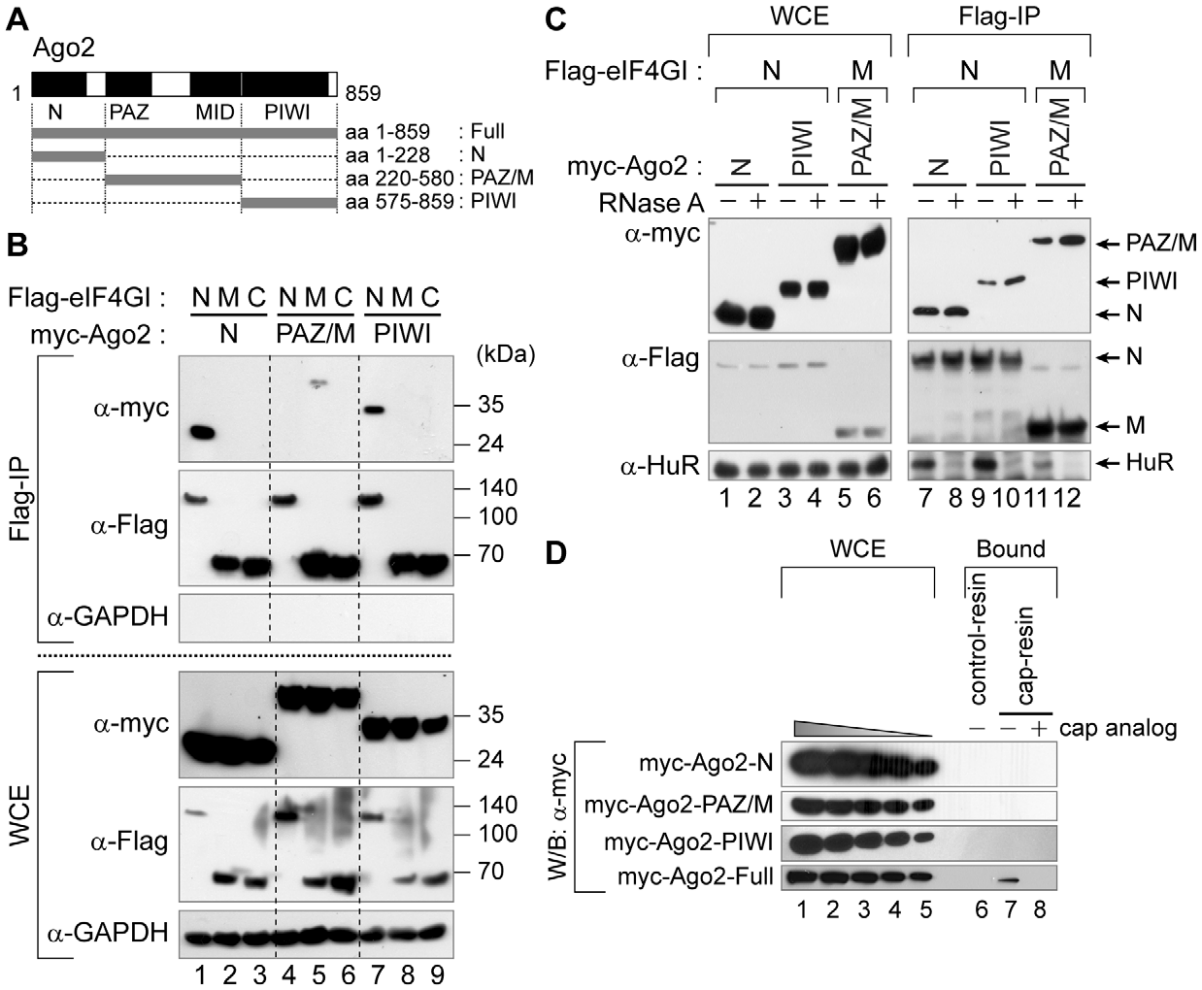
The intact Ago2 associates with the cap-binding complex. (*A*) A schematic diagram of human Ago2 and the constructs used for co-immunoprecipitation and cap-pulldown assays. (*B*) The domains in Ago2 required for the association with eIF4GI. 293FT cells were transfected with plasmids expressing Flag-tagged N-, M- or C-terminal portions of eIF4GI (Figure 5A) together with plasmids expressing myc-tagged Ago2 fragments corresponding to the N, PAZ/M or PIWI domains. Extracts from the transfected cells were incubated with the Flag-resin and the precipitated proteins were visualized by Western blotting using the indicated antibodies. (*C*) RNA-independent association of Ago2 domains with eIF4GI domains. RNase-treated (lanes 2, 4, 6, 8, 10 and 12) or -untreated (lanes 1, 3, 5, 7, 9 and 11) WCE from 293FT cells transiently expressing Flag-tagged eIF4GI derivatives (eIF4GI-N, -M or -C) with myc-tagged Ago2 variants (Ago2-N, -PAZ/M or -PIWI) were subjected to the immunoprecipitation experiments using the Flag-resins. The bound proteins were detected using antibodies indicated. (*D*) Association of Ago2 domains with the cap-resin. 2 mg of WCEs from 293FT cells transfected with plasmids expressing myc-tagged Ago2 derivatives were applied to cap-pulldown assays and the bound proteins were monitored using anti-myc antibodies. For comparison, various amounts corresponding to 2–0.4% of WCEs used in the pulldown assay were loaded in lanes 1–5.

Human Ago2 has several structural modules required for the miRNA-mediated gene silencing such as the N, PAZ, MID and PIWI domains [34]. Based on its structural features, we generated three Ago2 fragments representing the N, PAZ/M, and PIWI domains (aa 1–228, aa 220–580, and aa 575–859, respectively) (Figure 6A). To identify the domain(s) of Ago2 that participates in its association with eIF4GI, we co-transfected 293FT cells with plasmids expressing both Flag-tagged eIF4GI derivatives and myc-tagged Ago2 derivatives, and performed Flag-IP experiments. We found that the N and PIWI domains co-precipitated with the N-terminal domain of eIF4GI (Figure 6B, lanes 1 and 7 of the α-myc portion of the upper panel), whereas the PAZ/M fragment co-precipitated with the middle domain of eIF4GI (Figure 6B, lane 5 of the α-myc portion of the upper panel). And all of the co-precipitations were not affected by RNase treatment, indicating that these associations occur through RNA-independent manners (Figure 6C). These imply that each domain of Ago2 associates inter-independently with eIF4GI.

We further determined which part of Ago2 is responsible for its cap-association. For this purpose, we transfected 293FT cells with the plasmids encoding myc-tagged Ago2 derivatives and performed cap-pulldown assays (Figure 6D). None of Ago2 derivatives (N, PAZ/M and PIWI) except for the full-length form associated with the cap-resin, although they associated with eIF4GI in cells (Figure 6B). Our data suggest that the whole region of human Ago2 is needed for the association with the cap-binding complex. This result contradicts to the previous report that the MID domain of dAgo1 alone can bind to the cap-resin [17]. The discrepancy may result from the difference between the human and *Drosophila* Ago proteins or from the differences in experimental conditions.

### The 5′ cap structure may contact with Ago2

**Figure 7:**
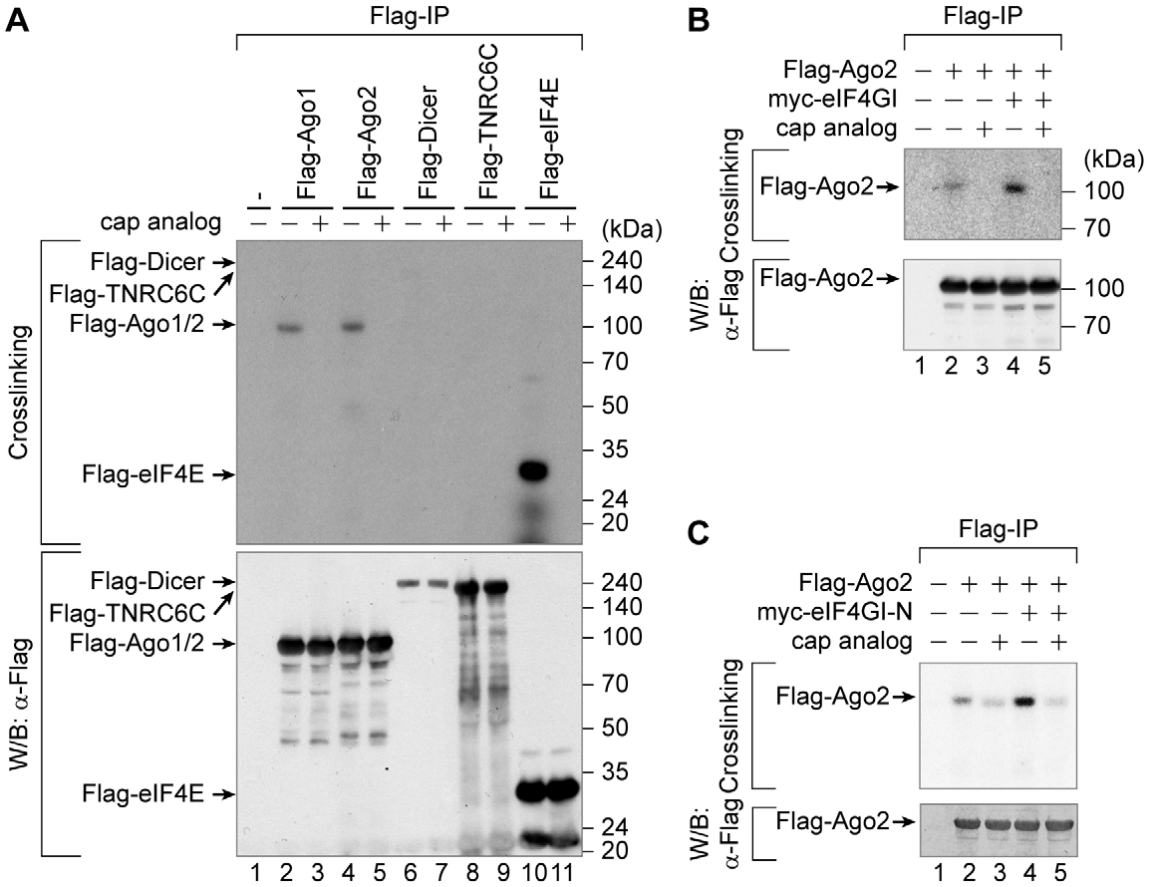
The 5′ cap structure may directly contact with Ago2. (*A*) WCEs (2 mg) from 293FT cells transfected with plasmids expressing Flag-Ago1, Flag-Ago2, Flag-Dicer, Flag-TNRC6C or Flag-eIF4E were subjected to Flag-IP, and cap-labeled RNAs were then incubated with the immunoprecipitated proteins in the presence or absence of cap analogs. The RNAs and proteins were photo-crosslinked by UV irradiation and then analyzed as described in the Materials and methods (upper panel). In parallel, Western blot analyses with an anti-Flag antibody were used to measure the amounts of the precipitated Flag-tagged proteins (lower panel). The positions of the proteins on the gel are denoted by arrows. (*B*) The effect of eIF4GI on the cap-crosslinking of Ago2. The resin-bound proteins from Flag-IP experiments using 293FT cell extracts expressing Flag-Ago2 and myc-eIF4GI were subjected to a cap-crosslinking assay (upper panel) and Western blotting (lower panel). (*C*) The effect of the N-terminal part of eIF4GI on the cap-crosslinking of Ago2. 293FT cells were co-transfected with plasmids expressing Flag-Ago2 and myc-tagged eIF4GI-N (aa 42–622), and a cap-crosslinking assay was performed.

Although we and others observed the cap-association of Ago by cap-pulldown assays, it has been controversial whether Ago directly interacts with the cap structure [11], [17], [19]. To validate an ability of the direct binding of Ago to the cap structure, we devised a method called a cap-crosslinking assay by modifying an *in vitro* RNAi assay [28]. We prepared cell extracts from 293FT cells transfected with plasmids expressing Flag-tagged Ago1, Ago2, Dicer, TNRC6C, or eIF4E. The Flag-tagged proteins and their associated proteins were purified using the Flag-resin. Capped RNAs were 5′-labeled with [α-^32^P]GTP using a vaccinia capping enzyme [35], incubated with the resin-associated Flag-tagged protein complexes, and then photo-crosslinked by UV irradiation. The unbound portions of the RNA were removed by RNase treatment, the beads were washed once with the lysis buffer, and the covalently-crosslinked RNA-protein complexes bound to the Flag-resin were resolved by SDS-PAGE. The proteins that were in direct contact with or very close to the cap structure could then be visualized by the autoradiography. For example, eIF4E, which directly interacts with the cap structure, showed a strong signal (Figure 7A, lane 10 in the upper panel) that disappeared when the resin and the cap-labeled RNAs were incubated with cap analogs as competitors (Figure 7A, lane 11 in the upper panel). Notably, although eIF4G co-precipitated with eIF4E through an apparent protein-protein interaction (Figure 2A, lane 10), their association was not detected in our cap-crosslinking assay (Figure 7A, lane 10 in the upper panel), suggesting that the cap-crosslinking happens when a protein is positioned very near to the cap structure. Indeed, a cap-crosslinking experiment using the Flag-eIF4GI-containing immunopurified complex from human cells showed no crosslinked pattern equivalent to the molecular weight of eIF4G, but it gave the crosslinked band for eIF4E (data not shown), similar to the previous reports [36], [37]. Importantly, both Ago1 and Ago2 proteins showed positive results in the cap-crosslinking assays (Figure 7A, lanes 2–5 in the upper panel). On the other hand, Dicer and TNRC6C, which are known to interact with Ago, showed negative results (Figure 7A, lanes 6–9 in the upper panel), meaning that they are not in direct contact with the cap structure. These results indicate that the Ago2 specifically interacts with or stay very close to the cap structure under certain conditions.

We further investigated the effect of eIF4GI on the cap-crosslinking of Ago2, and found that co-expression of eIF4GI with Ago2 in 293FT cells enhanced the cap-crosslinking of Ago2 (Figure 7B, compare lane 4 with lane 2 in the upper panel). This may indicate that eIF4GI facilitates the Ago2-cap association. We also tested whether the N-terminal region of eIF4GI (eIF4GI-N), which facilitates the cap-association of Ago2 (Figure 2), is sufficient to enhance the cap-crosslinking of Ago2. Ectopic expression of the eIF4GI-N augmented the cap-crosslinking of Ago2 (Figure 7C, compare lane 4 with lane 2 in the upper panel). Taken together, we conclude that Ago2 interacts with or localizes very close to the cap structure with the help of eIF4GI to facilitate the miRNA-mediated translational repression.

## Discussion

The Ago proteins are known to play key roles in the miRNA-mediated translational repression, but the molecular basis of the translational repression is unclear. To investigate the repression mechanism, we herein sought to identify a translation factor that can participate in the translational repression, particularly focusing on the Ago2-cap association. First, we used a cap-pulldown assay to investigate whether Ago2 can associate with the mRNA cap-binding complex in human cells. Both Ago1 and Ago2 specifically associated with the cap-resin (Figures 1A and 1C). Overexpression or knock-down of the eIF4GI affected the association of Ago2 with the cap-resin (Figures 2A and 2B), indicating that eIF4GI facilitates the Ago2-cap association. The N-terminal domain of eIF4GI (aa 42–622) was sufficient for the facilitation of Ago2-cap association, whereas its middle and C-terminal domains were not (Figure 2C). We then investigated the putative role of eIF4GI in the miRNA-mediated gene silencing. Knock-down of eIF4GI de-repressed the translational repression similarly to the knock-down of Ago2 (Figures 3D and 4D). These data collectively suggest that eIF4GI participates in the miRNA-mediated translational repression.

### How does eIF4GI facilitate the Ago2-cap association?

One possible scenario is through a protein-protein interaction between eIF4GI and Ago2. Co-immunoprecipitation experiments with Ago2 and eIF4GI deletion mutants indicated that the N-terminal end of eIF4GI (aa 42–202) is sufficient for its association with Ago2 (Figures 5D and 5E). However, it is insufficient for augmentation of the Ago2-cap association and the deletion of a region containing the aa 601–622 of eIF4GI abolished its capability of facilitating the Ago2-cap association (data not shown). Considering that this region encloses the eIF4E-binding motif, the cap-binding protein eIF4E seems to participate in the Ago2-cap association. Additional investigations are required to confirm an involvement of eIF4E in the miRNA-mediated translational repression.

It is worthy of note that co-immunoprecipitation of eIF4GI and Ago2 does not necessarily mean that these two proteins interact directly. We could not confirm their direct interaction because the purification of human Ago2 was not possible in our experimental conditions. Considering that the N-terminal region of eIF4GI (aa 42–202) contains the PABP-binding motif called the PAM1 (Figure 5A) motif and associates with Ago2 in an RNA-independent manner (Figure 5E), we cannot rule out the possibility that PABP may participate in the eIF4GI-Ago2 association since PABP directly interacts with the PAM2 motif of GW182 [38] and GW182 directly binds to Ago2 [39]. Indeed, we found that GW182 associates with the cap-binding complex in human cells (Figure 1A). Therefore, the eIF4GI-Ago2 association could occur via the consecutive connection of eIF4GI-PABP-GW182-Ago2, which is in agreement with a recent report suggesting that PABP participates in the miRNA-mediated translational repression through *in vitro* experiments using *Drosophila* cell extracts [40] as well as the miRNA-mediated deadenylation [38].

However, we could not observe augmentation of the Ago2-cap association by the overexpression of PABP (Figure 2A). Moreover, the knock-down of PABP did not show apparent de-repression effect on the miRNA-mediated translational gene silencing in our experimental conditions (Figure 3D, lane 5; Figure 4D, lanes 4, 8, 12 and 16), even though the miCXCR4-mediated translational repression assay system has been well reported to mimic the translational repression by miRNAs (Figures 3B, 3C, 4B and 4C) [6], [10], [24], [25]. In fact, several controversial data on the function of PABP in the miRNA-mediated repression have been published recently using *in vitro* assay systems with the cell extracts originated from *Drosophila* S2 cells [41], *Drosophila* embryos [40], or zebrafish embryos [42]. Particularly, the report using *Drosophila* S2 cell extracts exogenously supplemented with reporter-specific miRNA duplexes suggested that PABP is not essential for the miRNA-mediated translational repression [41]. On the contrary, the research using *Drosophila* embryo extracts utilizing the endogenous miRNAs for *in vitro* translational repression assay suggested that PABP is required for the miRNA-mediated silencing [40]. To explain the discrepancy, the authors suggested that the pre-loading step in the former could miss a critical function of PABP in the miRNA-mediated translational repression [40]. This indicates that the requirement of PABP can be undetected under certain conditions. Therefore, we speculate that we could not detect the de-repression effect of PABP knock-down because we used an exogenously introduced small RNA called miCXCR4 by transfection instead of endogenous ones such as let-7 and miR-122 in our experimental systems.

### The interaction of Ago2 with the cap structure

Finally, we investigated whether human Ago binds directly to the cap structure. Even though dAgo1 has been reported to interact directly with the mRNA cap structure via an allosteric regulation by miRNA [17], there is no evidence for the direct interaction between human Ago2 and the cap structure since purification of human Ago2 protein is very difficult if not impossible. To overcome such an obstacle, we developed a method to monitor the putative cap-binding capability of Ago by modifying an *in vitro* RNAi assay suitable for monitoring the slicer activity of Ago proteins *in vitro* [28]. Both Ago1 and Ago2 were shown to interact with or to localize very close to the cap structure by the cap-crosslinking method (Figure 7A). Moreover, the co-expression of full-length eIF4GI or its N-terminal region (aa 42–622) augmented the cap-crosslinking of Ago2 (Figures 7B and 7C). Interestingly, TNRC6C, a paralogue of GW182 protein, was not crosslinked with the 5′ cap-labeled RNAs (Figure 7A, lanes 8–9). This implies that the cap-crosslinking event occurs specifically in a protein(s) that directly interacts with or localizes very close to the cap structure.

### Possible role of the eIF4F-miRNP association in the miRNA-mediated translational repression

Based on our findings and the previous reports, we can envision how the miRNP complex might inhibit translation. First, the miRNP associates with the target mRNA according to the sequence complementarity. Second, the miRNP associates with the eIF4F complex, probably via the eIF4GI-Ago2 association or through the consecutive connection of eIF4GI-PABP-GW182-Ago2. The association between the miRNP and the eIF4F brings the cap structure closer to Ago. Third, the eIF4F-miRNP association may inhibit the translation-activator function of eIF4F. Two plausible mechanisms can be imagined. <1> According to the cap-competition hypothesis, the 5′ cap structure of the target mRNA, in interaction with eIF4E in the eIF4F complex, may be transferred to Ago, leading to inhibition of the cap-dependent translation directed by the eIF4F. <2> The association of the miRNP with the eIF4F may hinder the recruitment of the translational machinery (the 40S ribosome) to the cap structure by inhibiting one or more of the activities of eIF4GI. In other words, the eIF4GI-Ago2 association may block eIF4G's interaction(s) with other proteins (e.g., eIF4E, eIF4A or eIF3) or hinder the association of the 40S ribosome with the eIF3 complex. Additional studies are needed to fully elucidate the detailed mechanism(s) underlying the translational repression by the eIF4GI-Ago2 association. Recently, Fukaya and Tomari reported that dAgo1-RISC blocks the formation of the 48S pre-initiation complex presumably by inhibiting the eIF4A-dependent translation initiation in *Drosophila* [43]. This suggests that a step(s), in which eIF4A participates, is one of targets for the miRNA-mediated gene silencing. eIF4A is a component of eIF4F complex, which is assembled by eIF4E-eIF4GI-eIF4A interactions, recognizing the 5′ cap structure via eIF4E [13]. In this report, we showed that eIF4GI is required for the miRNA-mediated translational repression (Figure 3), and that the cap-association of Ago2 depends on eIF4GI (Figures 2 and 7). These data together with the Fukaya and Tomari's report may indicate that the eIF4F complex as a whole may participate in the miRNA-mediated translational repression. Furthermore, the Ago2-cap association may have additional functions related to the miRNA-mediated gene silencing. For example, the close positioning of the miRNP to the cap structure may help recruit a decapping complex to the cap structures of target mRNA, or it may block the formation of the elongation-competent 80S ribosome [32].

In conclusion, we presented several lines of evidence suggesting that human Ago closely associates with the mRNA cap structure and that its cap-association can be modulated by the association with human eIF4G. This study provides a foundation for understanding how the miRNP complexes could access the cap-binding complex (or the cap structure) and then directs the miRNA-mediated gene silencing at the step of translation initiation.

## Supporting Information

Table S1 **Oligonucleotides used for constructing the various plasmids.**
(DOC)

Table S2 **PCR primers used to construct the eIF4GI deletion mutants.** Restriction sites are underlined. Gray boxes denote the regions complementary to eIF4GI. White boxes depict the stop codons.
(DOC)

Table S3 **Combination of PCR primers to construct the eIF4GI deletion mutants.**
(DOC)

Table S4 **PCR primers used to construct the Ago2 deletion mutants.** Restriction sites are underlined. Gray boxes denote the regions complementary to Ago2. White boxes depict the stop codons.
(DOC)

Table S5 **Combination of PCR primers to construct the Ago2 deletion mutants.**
(DOC)

Table S6 **PCR primers used to construct the λN-Flag-Ago2 deletion mutants.** Restriction sites are underlined. Gray boxes denote the regions complementary to Ago2. White boxes depict the stop codons.
(DOC)

Table S7 **Combination of PCR primers to construct the λN-Flag-Ago2 deletion mutants.**
(DOC)

Table S8 **PCR primers used to clone the genes encoding various translation factors and TNRC6C.** To obtain eIF3c, eIF4E1 or PABP, eIF3c-flag-F and eIF3c-flag-R, eIF4E1-psk-F and eIF4E1-psk-R, eIF4E1-flag-F and eIF4E1-flag-R, or PABP-flag-F and PABP-flag-R primers were used for the PCR reaction. To clone the TNRC6 gene, primers TNRC6C-F and TNRC6C-R were used for the primary PCR, and primers TNRC6C-flag-F and TNRC6C-flag-R were used for the secondary PCR. Restriction sites are underlined. Gray boxes denote the regions complementary to eIF4GI. White boxes depict the stop codons.
(DOC)
