# Supplementary material for: eIF4GI Facilitates the MicroRNA-Mediated Gene Silencing
Source: PLoS One. 2013 Feb 7;8(2):e55725. doi: 10.1371/journal.pone.0055725 (PMC3567085; doi:10.1371/journal.pone.0055725)
Supplement: Table S1 — Oligonucleotides used for constructing the various plasmids. (DOC) [file pone.0055725.s001.doc]

| **Name** | **Sequence** (5′-nucleotide-3′) |
| --- | --- |
| **Flag-sense** | CTAGCCGCCACCATGGACTACAAAGACGATGACGGTGATTATAAAGATGATGACATCGATTACAAGGATGACGATGACA |
| **Flag-antisense** | AGCTTGTCATCGTCATCCTTGTAATCGATGTCATCATCTTTATAATCACCGTCATCGTCTTTGTAGTCCATGGTGGCGG |
| **Myc-sense** | CTAGCCGCCACCATGGAGCAGAAACTCATCTCTGAAGAGGATCTGA |
| **Myc-antisense** | AGCTTCAGATCCTCTTCAGAGATGAGTTTCTGCTCCATGGTGGCGG |
| **CXCR4-3×Bulge-sense** | GATCTCCGGAAGTTTTCACAAAGCTAACACCGGATCGCAAGTTTTCACAAAGCTAACACCGGATCGCAAGTTTTCACAAAGCTAACACCGGATCGCGGATCCATCGCGAT |
| **CXCR4-3×Bulge-antisense** | ATCGCGATGGATCCGCGATCCGGTGTTAGCTTTGTGAAAACTTGCGATCCGGTGTTAGCTTTGTGAAAACTTGCGATCCGGTGTTAGCTTTGTGAAAACTTCCGGA |
| **λN-sense** | CTAGCATGGACGCCCAGACCCGCCGCCGCGAGCGCCGCGCCGAGAAGCAGGCCCAGTGGAAGGCCGCCAACGGCGGCAGCGGCGGT |
| **λN-antisense** | CTAGACCGCCGCTGCCGCCGTTGGCGGCCTTCCACTGGGCCTGCTTCTCGGCGCGGCGCTCGCGGCGGCGGGTCTGGGCGTCCATG |
