# Supplementary material for: eIF4GI Facilitates the MicroRNA-Mediated Gene Silencing
Source: PLoS One. 2013 Feb 7;8(2):e55725. doi: 10.1371/journal.pone.0055725 (PMC3567085; doi:10.1371/journal.pone.0055725)
Supplement: Table S2 — PCR primers used to construct the eIF4GI deletion mutants. Restriction sites are underlined. Gray boxes denote the regions complementary to eIF4GI. White boxes depict the stop codons. (DOC) [file pone.0055725.s002.doc]

| **Name** | **Sequence** (5′-nucleotide-3′) |
| --- | --- |
| **4GI-Nt-F** | GGCC AA GCT TCG AAC ACG CCT TCT CAG C |
| **4GI-P-F** | GGCC AA GCT TCG CCC CAG ATT GCT CCC |
| **4GI-S-F** | GGCC AA GCT TCG ACT GCC TCC ACA CCC A |
| **4GI-M-F** | GGC CAA GCT TCG ATC TTT GCC AGT ATG CAG |
| **4GI-C-F** | GGCC AA GCT TCG CGA CTG AGC TGG GGC |
| **4GI-Nt-R** | TCC CCGCGG TCA TGG CTG GTT CAT CAA AAC |
| **4GI-P-R** | TCC CCGCGG TCA AGT GCG GGC CCC AGA |
| **4GI-S1-R** | TCC CCGCGG TCA TTC CAA GAC TGG GGA TG |
| **4GI-S2-R** | TCC CCGCGG TCA TGG AAC CGG TTT GCT AAG |
| **4GI-S3-R** | TCC CCGCGG TCA TTC CTC AGG TTG GGC AG |
| **4GI-S4-R** | TCC CCGCGG TCA CTC TCC TCC TTT CTC ACT |
| **4GI-S5-R** | TCC CCGCGG TCA GAA GGC ATC CAG AAG GT |
| **4GI-S-R** | TCC CCGCGG TCA CTG ATC TGA CTT ATA TTC A |
| **4GI-E-R** | TCC CCGCGG TCA GAA CTG AAA ACC AAG CAG |
| **4GI-M-R** | TCC CCGCGG TCA CCC TCC AGG TGC AAA G |
| **4GI-Ct-R** | TCC CCGCGG TCA GTT GTG GTC AGA CTC C |
