# Supplementary material for: eIF4GI Facilitates the MicroRNA-Mediated Gene Silencing
Source: PLoS One. 2013 Feb 7;8(2):e55725. doi: 10.1371/journal.pone.0055725 (PMC3567085; doi:10.1371/journal.pone.0055725)
Supplement: Table S3 — Combination of PCR primers to construct the eIF4GI deletion mutants. (DOC) [file pone.0055725.s003.doc]

| **Deletion mutant** | **Primer pair** |
| --- | --- |
| **eIF4GI-N** | 4GI-Nt-F + 4GI-E-R |
| **eIF4GI-M** | 4GI-M-F + 4GI-M-R |
| **eIF4GI-NM** | 4GI-N-F + 4GI-M-R |
| **eIF4GI-MC** | 4GI-M-F + 4GI-Ct-R |
| **eIF4GI-C** | 4GI-C-F + 4GI-Ct-R |
| **eIF4GI-NtP** | 4GI-Nt-F + 4GI-P-R |
| **eIF4GI-NtPS** | 4GI-Nt-F + 4GI-S-R |
| **eIF4GI-NtPS1** | 4GI-Nt-F + 4GI-S1-R |
| **eIF4GI-NtPS2** | 4GI-Nt-F + 4GI-S2-R |
| **eIF4GI-NtPS3** | 4GI-Nt-F + 4GI-S3-R |
| **eIF4GI-NtPS4** | 4GI-Nt-F + 4GI-S4-R |
| **eIF4GI-NtPS5** | 4GI-Nt-F + 4GI-S5-R |
| **eIF4GI-S** | 4GI-S-F + 4GI-S-R |
| **eIF4GI-PSE** | 4GI-P-F + 4GI-E-R |
