# Supplementary material for: eIF4GI Facilitates the MicroRNA-Mediated Gene Silencing
Source: PLoS One. 2013 Feb 7;8(2):e55725. doi: 10.1371/journal.pone.0055725 (PMC3567085; doi:10.1371/journal.pone.0055725)
Supplement: Table S4 — PCR primers used to construct the Ago2 deletion mutants. Restriction sites are underlined. Gray boxes denote the regions complementary to Ago2. White boxes depict the stop codons. (DOC) [file pone.0055725.s004.doc]

| **Name** | **Sequence** (5′-nucleotide-3′) |
| --- | --- |
| **Ago2-corr-full-gfp-F** | CCC AA GCT TGG TAC TCG GGA GCC GGC C |
| **Ago2-paz-gfp-F** | CCC AA GCT TGG TCA GCA ACA GCG TTT TAC |
| **Ago2-mid-a-gfp-F** | CCC AA GCT TGG AAC AAC ATC CTG CTG CC |
| **Ago2-n-gfp-R** | G GAATTC TCA CTG TGC CTT GTA AAA CGC T |
| **Ago2-m-gfp-R** | G GAATTC TCA GGG CAG CAG GAT GTT G |
| **Ago2-piwi-gfp-R** | G GAATTC TCA AGC AAA GTA CAT GGT G |
