# Supplementary material for: eIF4GI Facilitates the MicroRNA-Mediated Gene Silencing
Source: PLoS One. 2013 Feb 7;8(2):e55725. doi: 10.1371/journal.pone.0055725 (PMC3567085; doi:10.1371/journal.pone.0055725)
Supplement: Table S7 — Combination of PCR primers to construct the λN-Flag-Ago2 deletion mutants. (DOC) [file pone.0055725.s007.doc]

| **Deletion mutant** | **Primer pair** |
| --- | --- |
| **Ago2-Full** | Ago2-corr-full-Npep-F + Ago2-piwi-Npep-R |
| **Ago2-N** | Ago2-corr-full-Npep-F + Ago2-n-Npep-R |
| **Ago2-PAZ/M** | Ago2-paz-Npep-F + Ago2-m-Npep-R |
| **Ago2-PIWI** | Ago2-mid-a-Npep-F + Ago2-piwi-Npep-R |
