# Supplementary material for: eIF4GI Facilitates the MicroRNA-Mediated Gene Silencing
Source: PLoS One. 2013 Feb 7;8(2):e55725. doi: 10.1371/journal.pone.0055725 (PMC3567085; doi:10.1371/journal.pone.0055725)
Supplement: Table S8 — PCR primers used to clone the genes encoding various translation factors and TNRC6C. To obtain eIF3c, eIF4E1 or PABP, eIF3c-flag-F and eIF3c-flag-R, eIF4E1-psk-F and eIF4E1-psk-R, eIF4E1-flag-F and eIF4E1-flag-R, or PABP-flag-F and PABP-flag-R primers were used for the PCR reaction. To clone the TNRC6 gene, primers TNRC6C-F and TNRC6C-R were used for the primary PCR, and primers TNRC6C-flag-F and TNRC6C-flag-R were used for the secondary PCR. Restriction sites are underlined. Gray boxes denote the regions complementary to eIF4GI. White boxes depict the stop codons. (DOC) [file pone.0055725.s008.doc]

| **Name** | **Sequence** (5′-nucleotide-3′) |
| --- | --- |
| **eIF3c-flag-F** | CCC AAG CTT TCG CGG TTT TTC ACC ACC |
| **eIF3c-flag-R** | ATTT GCGGCCGC TCA GTA GGC CGT CTG AG |
| **eIF4E1-psk-F** | CT CTA GAG ATG GCG ACT GTC GAA CCG C |
| **eIF4E1-psk-R** | GG AAT TCC TTA AAC AAC AAA CCT ATT TTT AG |
| **eIF4E1-flag-F** | CCC AAG CTT GCG ACT GTC GAA CCG GA |
| **eIF4E1-flag-R** | ATTT GCGGCCGC TTA AAC AAC AAA CCT ATT TTT AG |
| **PABP-flag-F** | GG GGT ACC GCG GTT AAC CCC AGT GCC CCC AGC |
| **PABP-flag-R** | CG GGATCC CGGGT TAA ACA GTT GGA ACA CCG GTG G |
| **TNRC6C-F** | GAC ACT GAC TCT GCC TCC AAC TGT G |
| **TNRC6C-R** | TCG GCT CTC CTG GTG CTG ATG ATG |
| **TNRC6C-flag-F** | CCC AAG CTT GCT ACA GGG AGT GCC CAG G |
| **TNRC6C-flag-R** | ATTT GCGGCCGC CTA CAG GGA CTC CCC GCT G |
